# Morbillivirus Experimental Animal Models: Measles Virus Pathogenesis Insights from Canine Distemper Virus

**DOI:** 10.3390/v8100274

**Published:** 2016-10-11

**Authors:** Renata da Fontoura Budaszewski, Veronika von Messling

**Affiliations:** 1 Laboratório de Virologia, Faculdade de Veterinária, Universidade Federal do Rio Grande do Sul, Porto Alegre 91540-000, Brazil; renata.fontoura@ufrgs.br; 2 Veterinary Medicine Division, Paul-Ehrlich-Institut, Federal Institute for Vaccines and Biomedicines, Langen 63225, Germany

**Keywords:** morbillivirus genus, canine distemper virus, animal models, pathogenesis

## Abstract

Morbilliviruses share considerable structural and functional similarities. Even though disease severity varies among the respective host species, the underlying pathogenesis and the clinical signs are comparable. Thus, insights gained with one morbillivirus often apply to the other members of the genus. Since the *Canine distemper virus* (CDV) causes severe and often lethal disease in dogs and ferrets, it is an attractive model to characterize morbillivirus pathogenesis mechanisms and to evaluate the efficacy of new prophylactic and therapeutic approaches. This review compares the cellular tropism, pathogenesis, mechanisms of persistence and immunosuppression of the *Measles virus* (MeV) and CDV. It then summarizes the contributions made by studies on the CDV in dogs and ferrets to our understanding of MeV pathogenesis and to vaccine and drugs development.

## 1. Introduction

Morbilliviruses are enveloped, negative-stranded RNA viruses which cause a moderate to severe respiratory and gastrointestinal disease and long-lasting immunosuppression in their respective hosts. Despite the availability of a safe and cost-effective vaccine, the *Measles virus* (MeV) remains one of the leading causes of death among young children in developing countries [1]. MeV infections are also associated with neurological complications that may occur during the acute disease phase, but also many years later, due to long-term persistence in the central nervous system (CNS).

Since humans are the only reservoir for MeV, it is a promising target for global eradication, and a world-wide eradication campaign is ongoing [1]. The successful eradication of the closely related *Rinderpest virus* (RPV) in 2011 demonstrates the general feasibility of this approach [2]. However, decreasing adherence to vaccination in industrialized countries has resulted in a re-emergence of the disease. The increase in outbreaks in North America and Europe in recent years has led to these regions missing eradication targets, endangering the success of the overall campaign [3]. To develop new prophylactic and therapeutic strategies that support these efforts, a better understanding of MeV pathogenesis and immune interference is required.

**Figure 1:**
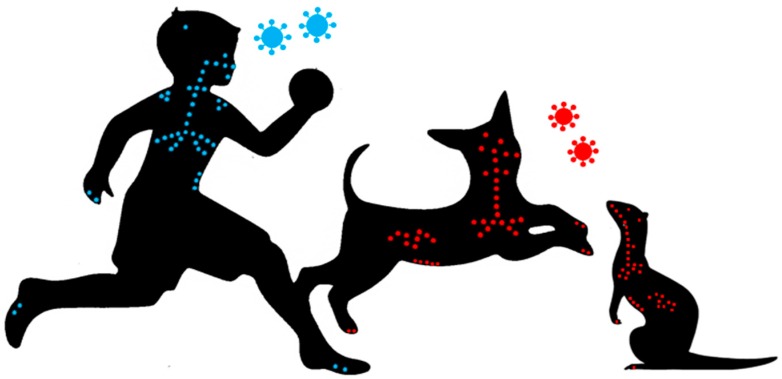
Morbilliviruses (*Measles virus* (MeV) in blue, *Canine distemper virus* (CDV) in red) display similar tropism and tissue distribution in their respective hosts.

While the narrow host range, which includes only humans and non-human primates, makes MeV eradication possible, it also limits the characterization of its pathogenesis. A surrogate model based on the study of the *Canine distemper virus* (CDV)—a closely related morbillivirus that infects a broad range of carnivores including ferrets or dogs—represents an attractive alternative. CDV causes a similar overall pathogenesis in its different hosts, but the disease severity varies from moderate in dogs, to completely lethal in highly susceptible species, such as ferrets and many wild carnivores [4,5]. The clinical signs include fever, often a characteristic rash, diarrhea, nasal discharge, conjunctivitis, and generalized immunosuppression, thereby reproducing the disease spectrum seen in MeV patients (Figure 1). Furthermore, acute and delayed neurologic complications are frequently observed [6]. This review will provide an update on the contribution of these models to our understanding of the routes of infection, receptors, tissue tropism, mechanisms of immunosuppression, and viral persistence in the CNS, and also an update on the progress that has been made in drug and vaccine development.

## 2. Morbillivirus Taxonomy and Life Cycle

**Figure 2:**
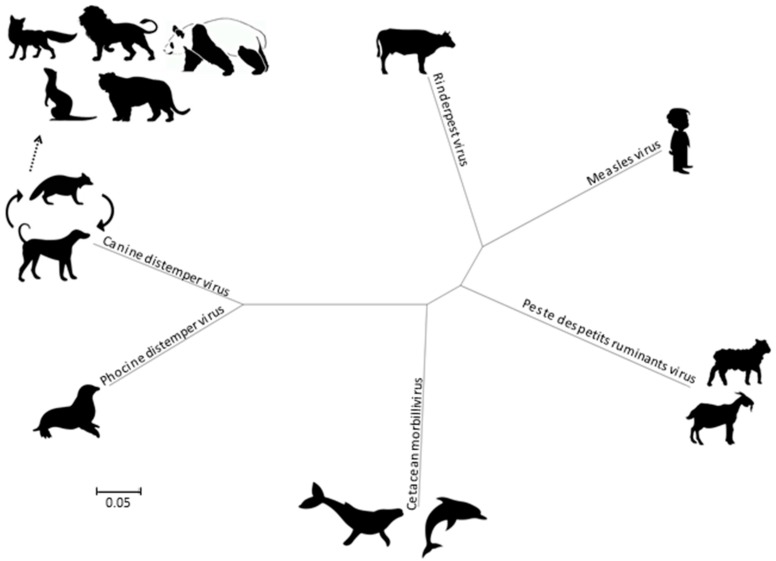
Phylogenetic tree, based on complete genomes of morbilliviruses. Molecular Evolutionary Genetics Analysis 6 (MEGA6) was used for phylogeny inference according to the maximum likelihood algorithm.

Morbilliviruses belong to the *Paramyxoviridae* family as a member of the *Mononegavirales* order. In addition to MeV, CDV, and RPV, the *Morbillivirus* genus currently includes four species, namely the *Cetacean morbillivirus* (CeMV), the *Phocine distemper virus* (PDV), the *Peste-des-petits-ruminant virus* (PPRV) and the recently added *Feline morbillivirus* (FeMV). Phylogenetically, the CDV and PDV are the most closely related (Figure 2), suggesting that the PDV emerged from the CDV several thousands of years ago by contact with terrestrial carnivores [7]. The CeMV forms a separate branch that includes isolates from dolphins and porpoises, while the MeV is most closely related to the ruminant morbilliviruses: RPV and PPRV. The RPV has been suggested as the progenitor virus of the *Morbillivirus* genus [8].

All morbilliviruses share the same particle structure and genome organization. Viral particles are pleomorphic, with a diameter of approximately 150 nm. They consist of a lipid envelope composed of the fusion (F) and attachment (H) transmembrane glycoproteins and are lined by the matrix (M) protein. The viral genomic RNA is encapsidated by the nucleoprotein (N), and forms, together with the phosphoprotein (P) and the polymerase (L) protein, the ribonucleoprotein-complex (RNP), which is surrounded by the viral envelope during the budding process at the plasma membrane [9]. The morbillivirus genome size lies at around 16,000 nt, and the genome contains six transcription units arranged linearly in the order 3’leader-N-P-M-F-H-L-5’trailer, which are separated by intergenic regions. The transcription units give rise to at least eight proteins, since the *P* gene also encodes the C and V proteins by use of an alternate open reading frame and RNA editing, respectively [10].

There is considerable homology on the amino acid and even nucleotide level, which results in high levels of structural and functional conservation among morbilliviruses. On the one hand, this allows the application of structural data available for MeV proteins to those of other morbilliviruses [11,12], and on the other hand, it enables the use of chimeric viruses or in vitro trans-complementation for the characterization of functional domains [13,14].

## 3. Pathogenesis

**Figure 3:**
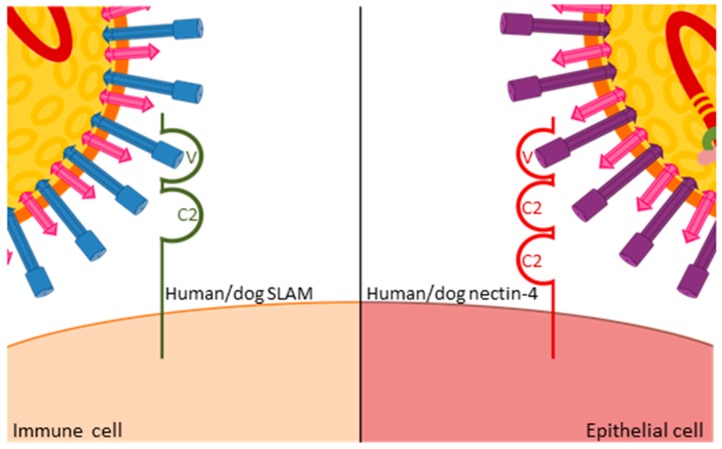
Similar interactions of MeV and CDV H proteins with the variable domain of human and dog CD150 receptors on immune cells, and human and dog nectin-4 receptors on epithelial cells, respectively.

All morbilliviruses are transmitted by aerosol. Taking advantage of eGFP-expressing viruses, resident immune cells in the respiratory tract were identified as initial target cells [15], and the subsequent spread to local immune organs and systemic dissemination coincides with a first fever peak observed three to six days after infection [16]. This stage of infection is critically dependent on the interaction of the virus with the immune cell receptor CD150 or signaling lymphocyte activation molecule (SLAMF1, [17]). CD150 has two extracellular immunoglobulin-superfamily domains, V and C2, and the cytoplasmic tail carries several tyrosine phosphorylation motifs involved in intracellular signaling [18,19,20]. Structure-function studies revealed that the H protein interacts with its variable domain (Figure 3), and that the residues involved in this interaction are located in close structural proximity in the MeV and CDV H proteins [11,21]. Mutation of these residues resulted in viruses that no longer infected immune cells but retained wild type entry and replication efficiency in epithelial cells. In ferrets, these SLAMblind viruses were unable to establish a systemic infection or cause clinical signs of disease [22], but elicited a protective immune response, suggesting that limited replication, possibly in epithelial cells close to the inoculation site, had occurred. A similar phenotype was observed in primates infected with SLAMblind MeV [23], further validating these findings.

The efficiency of immune recognition by the respective host determines the extent of virus amplification in immune cells and thereby the level of spread to epithelial cells. In ferrets, which are unable to control wild type viruses, infection levels exceeding 80% of B or T cells can be observed in certain immune tissues [24], whereas levels below 10% are usually found in MeV-infected macaques [25]. Consequently, there is a massive infection of epithelial tissues in wild type virus-infected ferrets, leading to severe respiratory and gastro-intestinal signs of disease and death within two to five weeks [16,26], whereas most primates develop only localized infection of epithelial cells and mild clinical signs [25]. Upon the identification of nectin-4—an adhesion protein that also contains immunoglobulin-like domains (Figure 3)—as a morbillivirus epithelial cell receptor [27,28,29,30], nectin-4blind viruses were generated that retained their ability to infect immune cells and resulted in wild type peripheral blood mononuclear cell (PBMC) infection levels and immunosuppression in ferrets. However, no rash or other clinical signs were observed, and no virus was shed [31]. The corresponding MeV in primates also remained immunosuppressive and was no longer shed [32], again illustrating the common role of receptor interactions in both viruses. Furthermore, studies with these selective receptor-blind viruses revealed that immunosuppression is caused by immune cell infection while clinical signs, and likely also transmission, result from epithelial cell infection. This link between specific target cell populations and defined clinical aspects of the disease provides starting points for therapeutic and outbreak control strategies.

## 4. Immunosuppression

The severe transient immunosuppression, which occurs at variable levels and can last from weeks to months after the resolution of the disease, is one of the hallmarks of morbillivirus infections. While a profound leukopenia during the acute infection phase is typical for many viral infections, morbilliviruses additionally induce an anergy-like state in immune cells that prevents their ex vivo activation by non-specific stimuli and leads to the loss of delayed-type hypersensitivity responses [33]. It is this long-term immunosuppression that leaves patients with an increased susceptibility to secondary infections such as pneumonia and gastroenteritis, which significantly contribute to MeV-associated morbidity and mortality.

**Figure 4:**
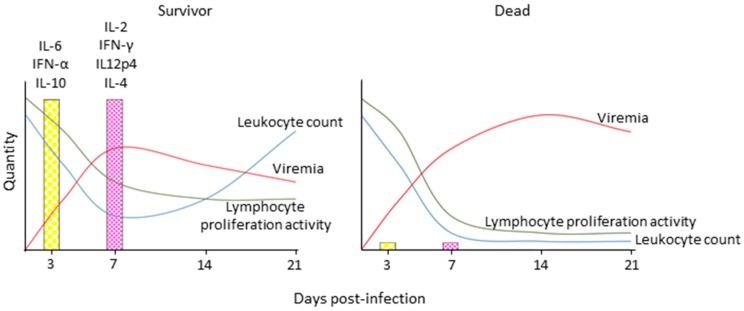
Schematic depiction of immune response profiles associated with different morbillivirus disease outcomes. Survivors display robust innate immune activation and experience transient immune suppression as indicated by a transient drop in leukocytes and lymphocyte proliferation activity upon non-specific stimulation, robust induction of innate and then adaptive cytokine responses, and control of cell associated viremia, while animals that succumb to the disease experience severe leukopenia and complete loss of lymphocyte proliferation activity, and are unable to activate innate immune responses and control the virus. IL: interleukin; IFN: interferon.

In CDV-infected dogs and ferrets and MeV-infected macaques, leukopenia is first observed two to four days after infection, when the replication in immune cells is just beginning; it peaks at the onset of clinical signs, and gradually resolves with the immune response-mediated control and elimination of the virus (Figure 4) [34,35,36]. It coincides with the depletion of immune cells from lymphatic tissues and an increase of cells in an early apoptotic state [24]. While leukocyte numbers normalize quickly after resolution of the disease, the anergic state persists over a prolonged period of time, which indicates that it is not only caused by a direct mechanism of immune cell destruction, but that indirect mechanisms are involved [37]. Studies in patients revealed a Th2-biased response, which, together with increased production of the immunosuppressive cytokine interleukin (IL)-10, may be involved in these alterations [38,39], but this phenomenon remains to be investigated in more detail in CDV or MeV animal models.

In contrast, the viral interference with innate immune activation has been extensively characterized. A comparison of cytokine profiles revealed a complete lack of innate immune activation in animals that succumbed to infection, whereas those that survived displayed a broad upregulation of cytokines associated with innate and later adaptive immune responses [40]. Consistent with many other members of the *Paramyxoviridae* family, the morbillivirus V protein inhibits innate immune signaling pathways on several levels [41,42]. Deletion of the V protein results in attenuation of lethal CDV wild type strains in ferrets, and loss of inhibition of lymphocyte proliferation, but the leukopenia is still observed [22]. A more detailed investigation of the contribution of different interferon (IFN) signaling pathways revealed that interference with signal transducer and activator of transcription 2 (STAT2) and melanoma differentiation-associated gene 5 (mda5) signaling was essential for effective immunosuppression and a lethal disease phenotype, while restored STAT1 signaling alone did not attenuate the virus sufficiently to lead to survival [43]. However, a STAT1blind MeV was attenuated in macaques [44], demonstrating a role of STAT1 signaling in Morbillivirus pathogenesis. Similarly, a C protein-deleted CDV retained full lethality in ferrets [22], while the corresponding MeV was clearly attenuated in macaques [45]. Both cases illustrate that, because of their high sensitivity to CDV, ferrets may thus not be ideally suited to characterize minor attenuation factors.

## 5. Neurologic Complications

MeV infection can lead to several rare but potentially lethal neurologic complications: acute disseminated encephalomyelitis (ADEM), also known as post-infectious encephalomyelitis (PIE); measles inclusion body encephalitis (MIBE); and subacute sclerosing panencephalitis (SSPE) [46]. In contrast, neuroinvasion is frequently observed during the acute phase of CDV infections, and dogs that survived a natural infection occasionally develop old dog encephalitis (ODE) that shares similarities with SSPE [47]. While around 30% of dogs infected with CDV will develop neurologic complications [48], certain strains cause close to 100% neuroinvasion in ferrets [26,49], making them an attractive model to characterize the underlying mechanisms.

ADEM, as a consequence of MeV infection, occurs in 1 in 1000 cases, and up to 25% of affected patients die, with around 33% of survivors experiencing chronic sequelae [46]. Only few infected cells are usually detected in the CNS, and an abnormal immune response to myelin basic protein is considered the primary pathomechanism [50]. In CDV-infected dogs, first infected cells are found at the interface between endothelial or epithelial cells and CNS cells around two to three weeks after infection, at the same time as epithelial cell infection is seen in other tissues [51]. If the virus is not cleared, focal infection of neurons and glial cells in the grey and white matter ensues four to five weeks after infection, with a concomitant onset of demyelination in these areas [52]. There is increasing evidence that this demyelination primarily results from an antiviral response in the CNS [53,54,55], even though virus-induced death of infected cells may also be a contributing factor [56]. While the presence of a virus in the CNS seen in CDV-infected animals clearly differs from the findings in ADEM patients, where little to no CNS infection is seen, the characterization of the immune mediated demyelination in this model may still provide valuable insights into the mechanisms involved in ADEM.

MIBE typically affects immunocompromised patients and is most often reported in HIV positive children, leukemia patients, and transplant patients undergoing immunosuppressive therapy [57,58]. The onset is usually within weeks or months after the acute infection, and the disease is characterized by behavioral changes and seizures [46]. Since ferrets are unable to mount an effective immune response to CDV infection, the neuroinvasion seen in that model likely reproduces key aspects of MIBE pathogenesis, especially cases with an onset during the acute phase. Time course studies in ferrets infected with neurovirulent strains revealed the importance of anterograde neuroinvasion via the olfactory bulb in addition to the previously described hematogenous spread, illustrating a direct entry route from the olfactory mucosa in the upper respiratory tract into the CNS [26,49,59]. The observation that the extent of immunosuppression determines not only disease duration but also the neurovirulence of the respective virus, further substantiates the link between the efficiency of the host immune response in controlling the infection and the incidence of neuroinvasion [60].

SSPE primarily affects patients infected in their first year of life and is exceedingly rare, with an incidence rate of around one in 10,000 cases [61,62]. It progresses slowly with demyelination of multiple areas of the brain. Initial symptoms include subtle behavioral changes, followed by myoclonic seizures leading invariably to death [46]. The mechanisms underlying this CNS persistence are poorly understood, but SSPE virus particle assembly is severely impaired due to hypermutations in the *M* and *F* gene regions [63,64]. While the pathogenesis of old dog encephalitis is similar, it is hitherto unknown if the virus accumulates similar hypermutations. Since only a small subset of animals that survive a CDV infection develop this complication, a canine model has not been pursued so far. Shortly after SSPE viruses were first isolated, their neurovirulence in ferrets was compared with wild type isolates, revealing that certain SSPE strains led to encephalitis in all intracerebrally inoculated animals, with one of the strains even causing CNS persistence of several months [65]. Further investigations demonstrated that the incidence of persistent infections could be increased if the animals received an MeV vaccine prior to inoculation, and that the histological changes and the antibody responses reproduced those seen in SSPE patients [66]. However, this model has not been further developed or used in recent years. Instead, an in vitro culture system of different CNS cell types is being increasingly used to study the mechanisms involved in morbillivirus CNS persistence and to explore therapeutic strategies [67,68,69].

## 6. Vaccine and Drug Development

The live-attenuated MeV vaccines licensed in the 1960s represent one of the most successful public health interventions and remain efficacious today. However, the exceptionally high MeV basic reproductive number (R_0_), close to 20, requires at least a 95% coverage in a population to prevent transmission [70]. Even though exact values have not been determined, the similarities among morbilliviruses make it highly likely that their R_0_ are also similar. CDV vaccines were first marketed around the same time as MeV vaccines and are now an integral part of the standard puppy vaccination schedule [71]. In countries with widespread vaccination, CDV cases in dogs have become rare, but a CDV eradication program is currently not feasible due to its diverse wildlife reservoir.

Morbillivirus vaccines have so far retained their efficacy, since the antigenic stability of these viruses is remarkably high compared to other RNA viruses, likely due to constraints on their glycoproteins [72]. Nonetheless, the development of a new generation of vaccines is actively ongoing to explore the potential of morbilliviruses as vaccine platforms, and, in the case of CDV, to increase safety for highly sensitive species. Morbilliviruses stably incorporate additional genes in their genome, and immunization with such a bi- or multivalent virus elicits humoral and cellular immune responses against the added proteins [73]. Strong immunogenicity of the first such MeV vaccine carrying a chikungunya virus antigen has been seen in a clinical phase I trial [74], and the efficacy of a leishmania antigen-expressing CDV has been demonstrated in dogs [75], illustrating the potential of this platform.

Even though the live-attenuated CDV vaccines are fully apathogenic in dogs, they can cause severe clinical distemper in highly sensitive species such as black-footed ferrets or other wild carnivores [76,77], highlighting the limitations of arbitrary attenuation. For those species, different vaccine approaches have been evaluated, including DNA vaccines [78], non-replication competent and replication-competent vector vaccines [79,80], as well as rationally attenuated vaccines [81,82]. A CDV F and H protein-expressing canarypox-based vaccine has been licensed for several years now, and has been successfully used in various wildlife species [83,84]. In addition, these studies have significantly contributed to our understanding of morbillivirus correlates of protection and the role of maternal immunity. The efficacy of DNA and vectored vaccines that express the CDV H protein alone revealed that immune responses against this protein are sufficient to protect against morbillivirus infections [85,86,87]. Many of these vectors also displayed superior immunogenicity in the presence of maternal antibodies compared to live-attenuated vaccines [88,89], providing strategies that may also become relevant for MeV eradication.

The resurgence of MeV in many industrialized countries has rekindled the interest in developing therapeutic strategies against these viruses, and the similar life cycles and availability of a sensitive small animal model make CDV an attractive surrogate system to assess the safety and efficacy of promising candidates. Furthermore, therapeutic interventions to control CDV infection may also be considered for valuable animals. Among the most promising candidates is an orally bioavailable small molecule inhibitor of the MeV polymerase that was able to rescue ferrets from lethal CDV challenge when given shortly after infection [90]. Strategies targeting viral entry or fusion, which also use small molecule inhibitors [91,92], as well as the first generation of host-targeting molecules [93], are reaching the state of preclinical testing, which will likely also involve one of the CDV surrogate models.

A meta-analysis of several studies has recently drawn attention to the potential of post-exposure passive immune transfer to reduce MeV-associated deaths [94], and a canine hyperimmune serum was marketed in Europe for CDV therapy. Finally, monoclonal antibody therapy might also yield promising results against morbilliviruses. Earlier proof-of-concept studies in mice demonstrate the efficacy of monoclonal antibodies directed against CDV glycoproteins [95], and a development of similar MeV-specific antibodies might become attractive as eradication progresses.

## 7. Conclusions/Perspectives

The study of the CDV in its natural hosts is a powerful complement to the investigation of MeV in non-human primates or rodent models and has significantly contributed to our understanding of morbillivirus pathogenesis. The high sensitivity of ferrets to the CDV infection enables the identification of virulence and attenuation determinants, as well as the safety and efficacy assessment of new therapeutics and vaccines. The naturally occurring progressive CNS infection in dogs surviving the acute CDV infection provides unique insights into the pathogenesis of this devastating complication and may yield novel therapeutic strategies. Since dogs, as companion animals, develop many of the same cancers and degenerative disorders as seen in people, they also represent attractive translational models for morbillivirus-based gene therapy approaches. The comparative analysis in different virus-host models will be essential to answer still-open questions about antigenetic stability, immunosuppression and persistence and will continue to improve our understanding of these important viruses.
